# Supracerebellar Infratentorial Endoscopic and Endoscopic-Assisted Approaches to Pineal Lesions: Technical Report and Review of the Literature

**DOI:** 10.7759/cureus.1329

**Published:** 2017-06-09

**Authors:** Rita Snyder, Daniel R Felbaum, Walter C Jean, Amjad Anaizi

**Affiliations:** 1 Neurosurgery, Medstar Georgetown University Hospital

**Keywords:** endoscopic surgery, microscopic surgery, supracerebellar infratentorial, pineal, pineal gland cyst

## Abstract

The pineal gland has a deep central location, making it a surgeon’s no man’s land. Surgical pathology within this territory presents a unique challenge and an opportunity for employment of various surgical techniques. In modern times, the microsurgical technique has been competing with the endoscope for achieving superior surgical results. We describe two cases utilizing a purely endoscopic and an endoscopic-assisted supracerebellar infratentorial approach in accessing lesions of the pineal gland. We also discuss our early learning experience with these approaches.

## Introduction

Originating from the posterior external wall of the third ventricle, and superficial to the habenular and posterior commissures, the pineal gland is bordered laterally by the thalamic pulvinar nuclei, superiorly by the suprapineal recess and cerebral tentorium, and inferiorly by the apex of the cerebellar vermis and precentral venous complex. The tectum and fourth cranial nerves lie inferior to the gland, coursing anteriorly. Neoplasms of this region typify less than one percent of all adult intracranial lesions [1]. They may manifest with the signs and symptoms characteristic of obstructive hydrocephalus, compression of the cerebellar or midbrain structures. Classically, they may present with Parinaud syndrome. Pineal cysts are worth a specific mention. They can sometimes be difficult to discern from a neoplastic process. Importantly, they can be relatively common with a 25% - 40% incidence rate at autopsy. Large symptomatic cysts have a 3:1 predilection for the female gender, with a mean presentation age of 30 years [2]. 

Preoperative work-up includes full craniospinal contrasted magnetic resonance imaging (MRI), and obtaining serum and cerebrospinal fluid (CSF) levels of oncoproteins beta-human chorionic gonadotropin (B-hCG), alpha-fetoprotein (AFP), and placental alkaline phosphatase (PLAP) with cytology [3]. Tissue diagnosis is mandatory and can be obtained via several acceptable surgical routes. Ahmed, et al. cite a sampling error of 15% and recommend respective caution when interpreting results definitively [1]. Obtaining multiple samples to ensure an accurate diagnosis is essential. A potentially prudent approach is a transventricular endoscopic approach for a biopsy with simultaneous management of the hydrocephalus by third ventriculostomy [4]. Germ cell tumors, once diagnosed, are often treated with radiotherapy and chemotherapy. Once a surgical indication is confirmed, various anatomical approaches to these lesions may be considered. Classic skull base approaches include: supracerebellar infratentorial (SCIT), occipital interhemispheric transtentorial, and posterior interhemispheric transcallosal [5-6]. Certain approaches may employ an open microsurgical, endoscopic, or combined technique [7-8]. Uschold, et al. cite improved visualization and accuracy with the endoscope-assisted SCIT approach. It offers similar access to the pineal region as with a microscopic approach, but utilizes a smaller craniotomy [8]. 

Historically, indications for pineal cyst removal were limited to obstructive signs and symptoms such as hydrocephalus or brainstem compression. However, a recent retrospective study has shown surgery to be a successful treatment option for patients with non-specific symptoms (concurrent with the presence of a pineal cyst) on imaging. The authors concluded that episodic, positional, paroxysmal, or refractory chronic headaches, visual disturbances, and unexplained episodic loss of consciousness may benefit from pineal cyst resection. This is thought to be due to the relief of intermittent obstruction of the cerebral aqueduct. The mean cyst diameter was 1.5 cm among 18 patients (range 0.9 cm to 2.2 cm); all underwent either SCIT or occipital interhemispheric transtentorial pineal cyst removal. Out of 18 patients, 17 reported relief or improvement of symptoms [9]. We must note, as did the authors, that patient selection is key.

### Case 1: 

A 30-year-old male presented with seizure associated with several months of intractable headaches and diplopia. The work-up was significant for a pineal lesion causing brainstem compression. He underwent an uneventful endoscopic-assisted SCIT approach with total resection of pineocytoma. The patient recovered uneventfully with complete resolution of his related symptoms. 

### Case 2:

A 1.3 cm by 1.3 cm pineal lesion presented as a potential source of worsening positional headaches and retro-orbital pain refractory to conservative measures over a two year period. The 36-year-old female’s initial MRI imaging revealed evidence of a pineal cyst, and she underwent an endoscopic paramedian SCIT approach for cyst resection. The pathology of the specimen was consistent with a pineal cyst. The patient was discharged on postoperative day 2 following an uneventful hospital course. At three-month follow-up, preoperative ocular pressure and pain were resolved.

## Technical report

**Figure 1:**
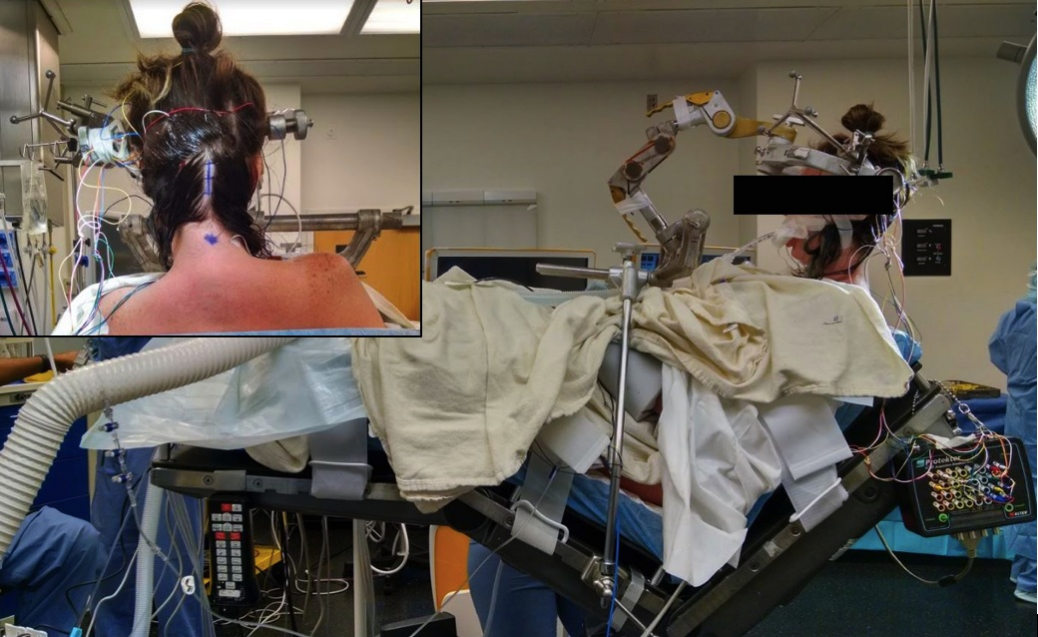
Patient positioning and incisional marking

The patient is placed in a sitting position with gentle neck flexion and the head is fixated using Mayfield pins (Figure 1). Post-positioning somatosensory potentials provided intraoperative monitoring. Intraoperative frameless stereotactic navigation was registered and aided in craniotomy planning.

**Figure 2:**
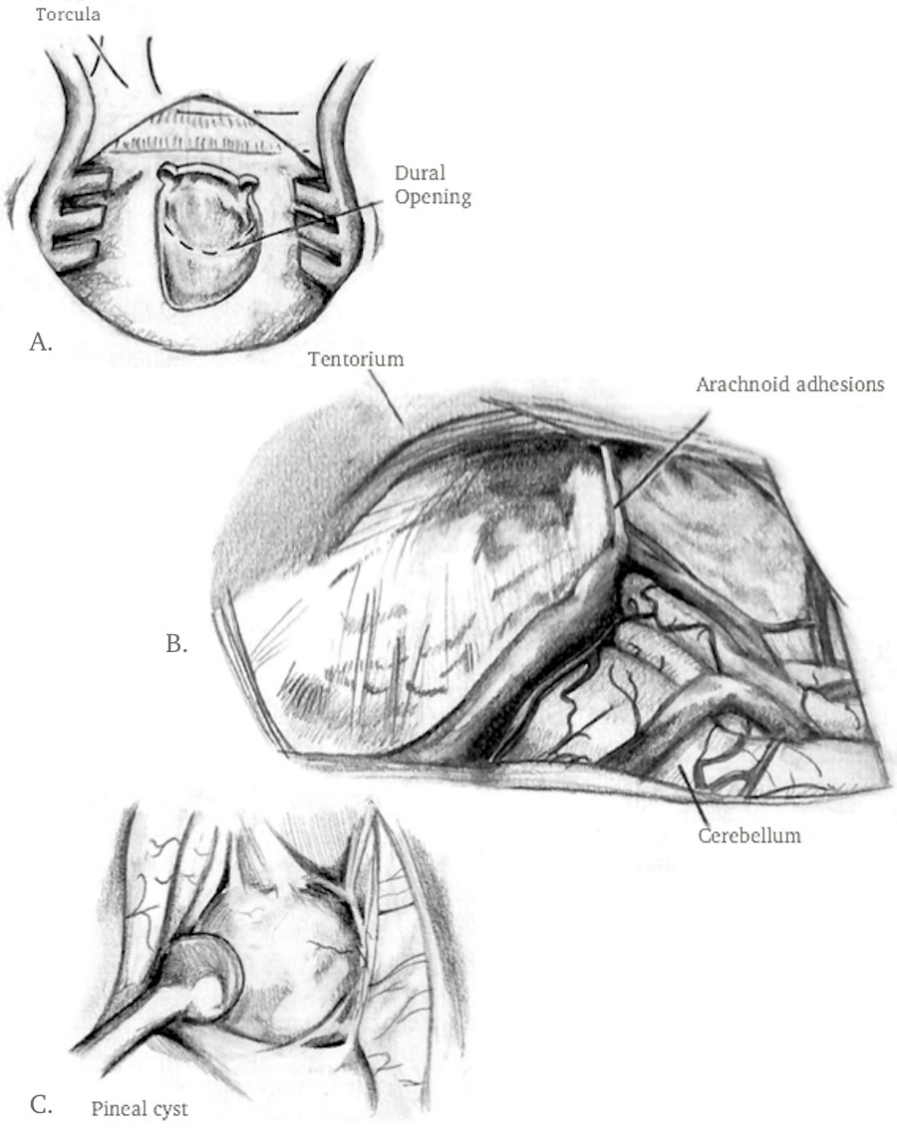
Artist's rendering of surgical exposure A) craniotomy and dural incision B) intraoperative endoscopic view of supracerebellar infratentorial cavity C) pineal cyst

**Figure 3:**
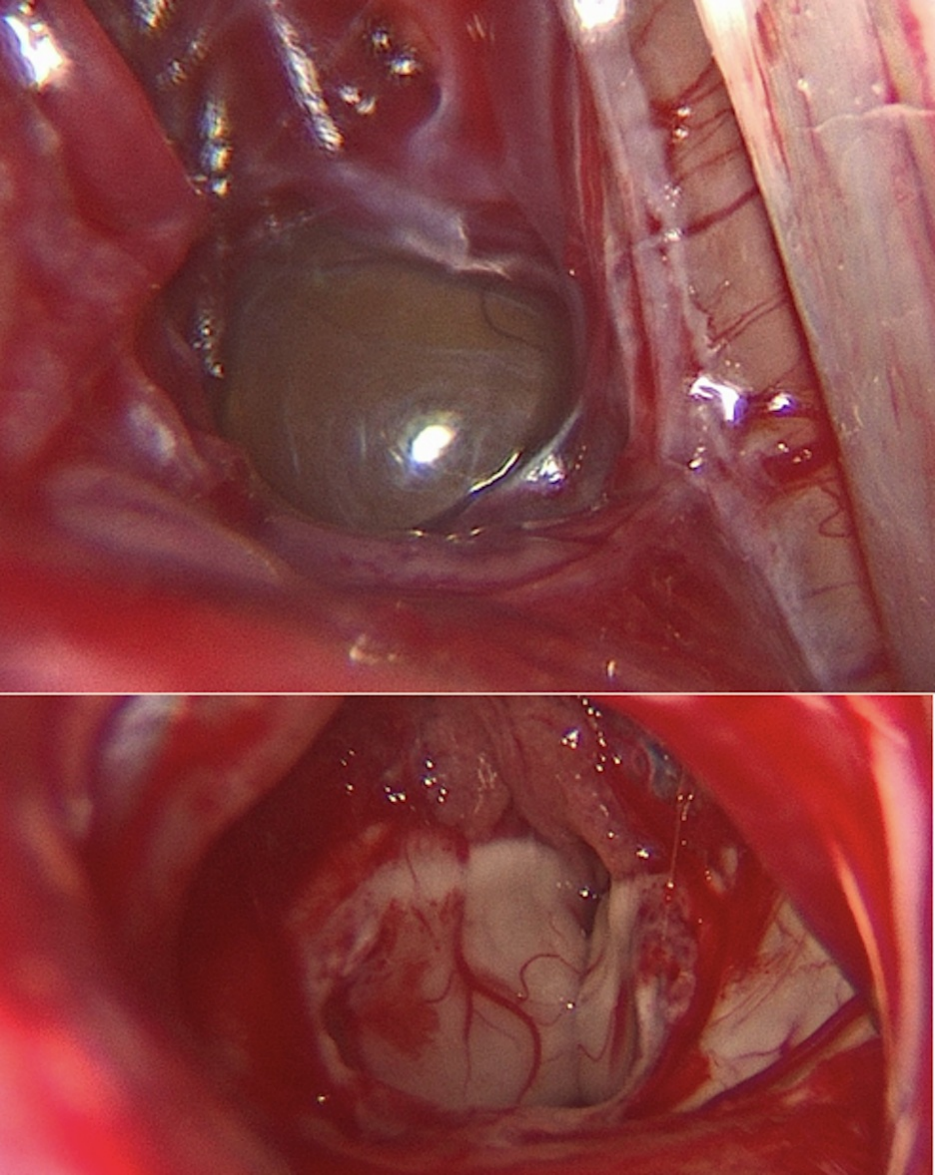
Pre- and post-resection endoscopic images

**Figure 4:**
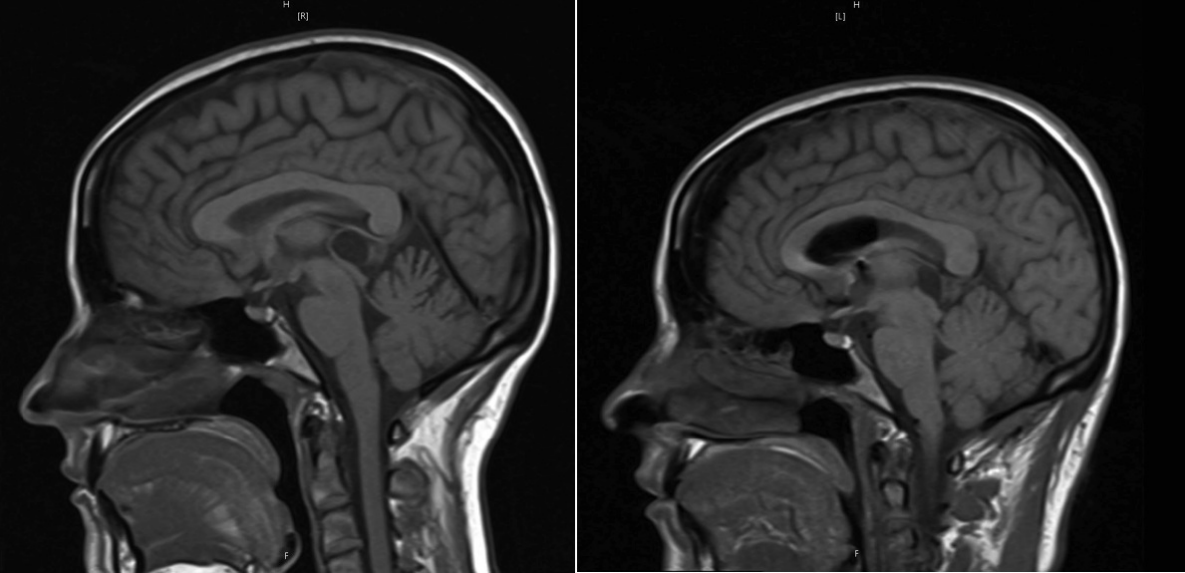
Pre- and post-operative T1-weighted magnetic resonance imaging

A right-sided off-midline suboccipital approach was used to gain access to the infratentorial space. A 2 cm x 2 cm bone flap was elevated. The dura was incised in a curvilinear fashion and reflected superiorly toward the transverse sinus (Figure 2A). Following the release of CSF to achieve cerebellar relaxation, the endoscope was introduced into the supracerebellar corridor (Figure 2B). Due to minimal bridging veins on the right side (seen in pre-operative MRI), a single superior bridging cerebellar vein required cautery. The endoscope provided full visualization of the posterior splenium and access to the pineal cyst (Figure 2C). Intraoperative navigation assisted with directing the endoscope. Upon exploration of the infrasplenial region, the cyst dome was located and meticulously dissected using a ball probe, and removed using a pituitary biter (Figure 3). Hemostasis was obtained and the craniotomy was closed in the standard fashion. The patient had an uneventful hospital course. Postoperative MRI revealed gross total resection (Figure 4).

## Discussion

Endoscopy has proven to be a successful technique for the treatment of skull base lesions previously considered inaccessible or fraught with complications. This technique now affords access to the ventral skull base, middle cranial fossa, clivus, petrous bone, infratemporal fossa, and pterygopalatine fossa [10]. Similar to the initial concerns with endonasal approaches, there is a similar controversy over endoscopic approaches to the pineal region. While the first endoscopic endonasal surgery was performed in 1909, it required recent technological developments to become a feasible and reliable alternative. The current “four hands, two nostrils” technique offers the lowest rates of infection and comorbidities associated with trans-sphenoidal endoscopy, minimizes entry trauma, and affords greater area of exposure and surgical freedom [11]. A recent systematic review and meta-analysis found endoscopy to be associated with higher gross total tumor resection, lower rate of septal perforation in treating pituitary adenomas, and lower incidence rates of residual tumor in comparison to microsurgery [12]. Additionally, a recent multicenter study has demonstrated endoscopy to have equal outcomes to microscopy, in the treatment of pituitary adenomas, despite a vast difference in surgeon experience (100 independent cases versus 1800) [13]. Drawbacks to trans-nasal endoscopy are similar to endoscopic approaches to the pineal region. One concern is difficulty in establishing proximal and distal control in cases of vascular insult [14]. Another concern is being able to attain hemostasis in a narrow operative corridor. Furthermore, there may be a potential need for two surgeons with a steep learning curve. Advances in neuronavigation, 3D binocular neuroendoscopy, robotics, and virtual reality training will continue to develop, ideally addressing these problems. 

The fully endoscopic approach in our case provided a less invasive access to the pineal region by taking advantage of the natural skull base corridors. In a prior report by our group, our experience with the endoscope has shifted from being used as an adjunct to being the sole approach necessary to this region [15]. This is similar to other institutional experiences [8, 16]. A keyhole craniotomy is all that is needed to provide maximal access with minimal disruption to the normal anatomy. A stereotactic intraoperative frameless navigation aided our approach. A well-planned paramedian craniotomy obviates the need to navigate the torcula and occipital sinus, and can help in avoiding the bridging and the precentral supracerebellar veins, which were managed to be left intact. In addition to minimizing the need for cerebellar retraction, the approach presented a less invasive view of the midline galenic venous complex, similar to other reports [8, 17].

## Conclusions

The purely endoscopic and endoscope-assisted paramedian supracerebellar infratentorial approch was successful in providing wide surgical navigability through a tight anatomical corridor. In appropriately selected patients, the endoscope can offer a less invasive option with improved visability compared to traditional skull base techniques. Ideally, future studies will further elaborate the indications, the advantages and disadvantages of purely endoscopic approaches in the treatment of pineal lesions. This may include cadaver studies demonstrating respective surgical parameters, and multicentre studies comparing operative times, complication rates, and overall outcomes.
